# Do Autism Spectrum and Autoimmune Disorders Share Predisposition Gene Signature Due to mTOR Signaling Pathway Controlling Expression?

**DOI:** 10.3390/ijms22105248

**Published:** 2021-05-16

**Authors:** Ekaterina A. Trifonova, Alexandra I. Klimenko, Zakhar S. Mustafin, Sergey A. Lashin, Alex V. Kochetov

**Affiliations:** 1 Federal Research Center Institute of Cytology and Genetics, Siberian Branch of the Russian Academy of Sciences, 630090 Novosibirsk, Russia; klimenko@bionet.nsc.ru (A.I.K.); MustafinZS@bionet.nsc.ru (Z.S.M.); lashin@bionet.nsc.ru (S.A.L.); ak@bionet.nsc.ru (A.V.K.); 2 Natural Science Faculty, Novosibirsk National Research State University, 630090 Novosibirsk, Russia

**Keywords:** mTOR, autism spectrum disorder (ASD), autoimmune disorders (AID), bioinformatics, SFARI Gene database, FMRP, vitamin D3

## Abstract

Autism spectrum disorder (ASD) is characterized by uncommon genetic heterogeneity and a high heritability concurrently. Most autoimmune disorders (AID), similarly to ASD, are characterized by impressive genetic heterogeneity and heritability. We conducted gene-set analyses and revealed that 584 out of 992 genes (59%) included in a new release of the SFARI Gene database and 439 out of 871 AID-associated genes (50%) could be attributed to one of four groups: 1. FMRP (fragile X mental retardation protein) target genes, 2. mTOR signaling network genes, 3. mTOR-modulated genes, and 4. vitamin D3-sensitive genes. With the exception of FMRP targets, which are obviously associated with the direct involvement of local translation disturbance in the pathological mechanisms of ASD, the remaining categories are represented among AID genes in a very similar percentage as among ASD predisposition genes. Thus, mTOR signaling pathway genes make up 4% of ASD and 3% of AID genes, mTOR-modulated genes—31% of both ASD and AID genes, and vitamin D-sensitive genes—20% of ASD and 23% of AID genes. The network analysis revealed 3124 interactions between 528 out of 729 AID genes for the 0.7 cutoff, so the great majority (up to 67%) of AID genes are related to the mTOR signaling pathway directly or indirectly. Our present research and available published data allow us to hypothesize that both a certain part of ASD and AID comprise a connected set of disorders sharing a common aberrant pathway (mTOR signaling) rather than a vast set of different disorders. Furthermore, an immune subtype of the autism spectrum might be a specific type of autoimmune disorder with an early manifestation of a unique set of predominantly behavioral symptoms.

## 1. Introduction

Autism spectrum disorder (ASD) is a heterogeneous, behaviorally defined, neurodevelopmental disorder with complex genetic and environmental components. It is still not clear enough whether autism comprises a vast set of different disorders, similar to intellectual disability, or a few disorders sharing common aberrant pathways. The search for genetic factors underlying ASD has led to the identification of about one thousand genes cataloged in the SFARI (Simon’s Foundation Autism Research Initiative) Gene database that has scored and ranked genes into four categories [1]. The syndromic category includes a number of genetic syndromes that manifest ASD at higher than expected frequencies compared to the general population. A substantial part of both syndromic and idiopathic autism cases can be attributed to disorders caused by mTOR-dependent translation deregulation [2,3,4].

The mechanistic target of rapamycin (mTOR) is a serine/threonine kinase and a key translation regulator, the central component of two multiprotein complexes—mTORC1 and mTORC2—which differ in protein compositions and ranges of substrates. mTOR signaling has been found to have a pathogenic role in a myriad of neurological disorders, such as epilepsy, autism, intellectual disability, dementia, traumatic brain injury, brain tumors, and hypoxic-ischemic injury [5].

At the same time, there is a growing appreciation of the mTOR pathway in adaptive immunity for its crucial roles in balancing T cell quiescence and activation. Under steady-state circumstances, mTOR is subtly inhibited by multiple mechanisms to maintain normal T cell homeostasis. Antigen recognition by naive T cells leads to mTOR activation, which subsequently promotes the differentiation of these cells into distinct effector T cell subsets [6]. Consistent with the use of rapamycin as an immunosuppressant, dysregulated mTOR signaling is involved in a variety of autoimmune diseases (AID). mTOR inhibition with rapamycin showed immunoregulatory effects in several autoimmune disorders, such as systemic lupus erythematosus (SLE) [7,8], chronic immune thrombocytopenia (ITP) [9], type 1 diabetes [10], experimental autoimmune encephalomyelitis (EAE) [11], and Crohn’s disease [12]. Inhibition of mTOR by everolimus has shown moderate efficacy in reducing joint inflammation in rheumatoid arthritis patients [13].

The uncommon genetic heterogeneity associated with ASD is quite unusual for such a highly heritable disorder. The concordance rates in monozygotic and dizygotic twins allows the estimation of the weight of the environment in determining disease susceptibility; by means of twin studies it was found that genetic factors play a consistently larger role than environmental factors [14]. However, familial risk and heritability of ASD may vary by cognitive ability. In the population-based cohort study, the broad-sense heritability for ASD − ID and ASD + ID were 64.6 (46.0–100.0%) and 33.4% (14.4–58.4%), respectively. The findings suggest that ASD − ID may have a greater genetic basis than ASD + ID, and ASD + ID heritability is relatively not very high [15].

Among the generally accepted opinions of autoimmune and chronic inflammatory diseases, there is now an agreement that these are complex conditions with the individual genetic predisposition providing a rate of heritability. Twin studies have been also performed in several AID and cumulatively suggest that some diseases, e.g., celiac disease and psoriasis, are highly genetically determined, while rheumatoid arthritis or systemic sclerosis have a limited role of genetics. Thus, several AID have extremely high concordance rates in monozygotic twins, specifically of psoriasis up to 72%, type 1 diabetes up to 64%, and hypothyroidism up to 55% [16]. 

Despite the relatively high level of heritability, most autoimmune disorders, similarly to ASD, are characterized by impressive genetic heterogeneity [17,18]. Previously, we have identified a unique signature of autism predisposition genes by dividing them into four categories: 1. FMRP target genes (targets of RNA-binding protein FMRP, a negative regulator of translation initiation, one of the key components of the local translation system), 2. whole mTOR signaling network genes, 3. mTOR-modulated genes (a subset of genes whose mRNAs appears to be exceptionally sensitive to changes in mTOR activity), and 4. vitamin D3-sensitive genes (a subset of genes whose expression is regulated by vitamin D3) [19]. To assess the genetic similarity between ASD and AID, we quantified the percentages of the above categories among the genes associated with multiple sclerosis (MS), systemic lupus erythematosus (SLE), juvenile rheumatoid arthritis (JRA), Crohn’s disease (CD), ulcerative colitis (UC), and type 1 diabetes (T1D).

## 2. Results

### 2.1. SFARI Gene Database and AID Genes Comparative Gene-Set and Pathway Analysis

Here, we have shown that 584 out of 992 genes included in the SFARI Gene database and 439 out of 871 autoimmune genes could be attributed to one of the four groups: 1. FMRP target genes, 2. mTOR signaling network genes, 3. mTOR-modulated genes, and 4. vitamin D3-sensitive genes (see Table 1). The complete list of SFARI Gene database and autoimmune genes divided into the above categories is given in Supplementary Table S2.

It is worth noting that, with the exception of FMRP targets, that are associated with the direct involvement of local translation disturbance in the pathological mechanism of ASD, the remaining categories are represented among AID genes in a very similar percentage as among ASD predisposition genes. Thus, mTOR signaling pathway genes make up 4% of ASD and 3% of AID genes, mTOR-modulated genes—31% of both ASD and AID genes, and vitamin D-sensitive genes—20% of ASD and 23% of AID genes. 

**Figure 1:**
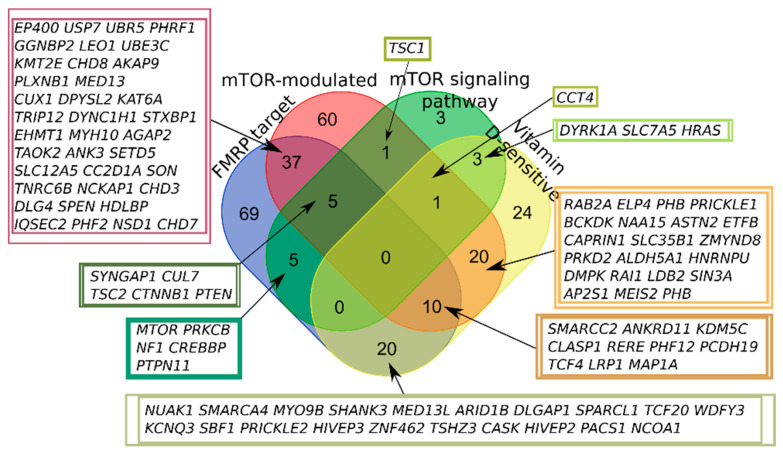
Venn diagram representing the relationship of the four categories related to the mTOR signaling and vitamin D-sensitive genes. All sets of genes were preliminarily intersected with high-scored candidates from the SFARI Gene database.

According to the previous SFARI Gene database scoring system, more than half of all scored genes were placed within the “Minimal Evidence Category”. Formerly, we have analyzed the first three categories of the SFARI Gene database (“high confidence”, “strong candidate”, and “suggestive evidence”) containing 281 genes in total. We found that 179 out of 281 genes (63.7%) could be included in one of the four groups [19]. Nowadays, the SFARI Gene database gene scoring system has been changed (https://gene.sfari.org/announcing-the-new-sfari-gene-and-gene-archive/ accessed on 12 November 2020) and the first two categories (“high confidence”, “strong candidate”) in the new system contain 401 genes in total (12-11-2020 release, Supplementary Table S1). We found that 258 out of 401 genes (64.3%) could be attributed to one of the four groups (Figure 1), and 143 did not belong to any of the selected categories. Thus, the increased number of high-scored SFARI genes did not fundamentally affect the pathway composition of the genes.

None of the high-scored SFARI genes fell into all four categories; the largest number of intersections was observed for FMRP target and mTOR-modulated genes (Figure 1).

**Figure 2:**
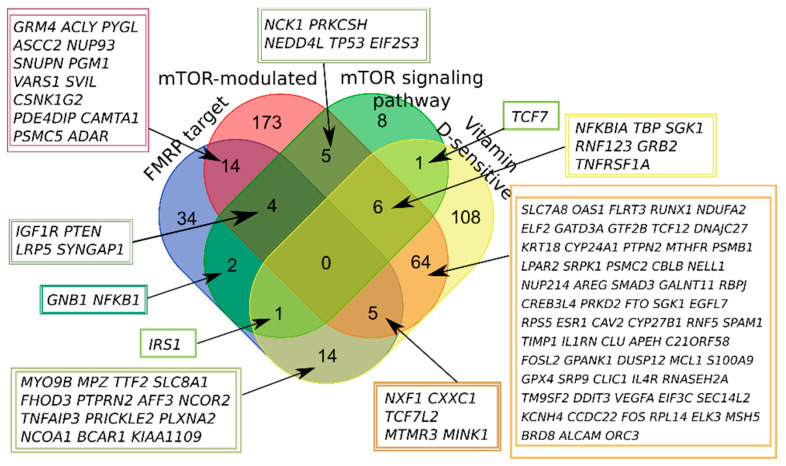
Venn diagram representing the relationship of the four categories related to the mTOR signaling and vitamin D-sensitive genes. All sets of genes were preliminarily intersected with whole AID gene set.

A significant portion of AID genes belonged to more than one of four categories, with the largest number of intersections observed for vitamin D-sensitive and mTOR-modulated genes, but it could be attributed to the number of genes in the categories (Figure 2). Thus, we first characterized 50.4% of autoimmune genes by dividing them into categories associated with the mTOR signaling pathway and vitamin D.

**Figure 3:**
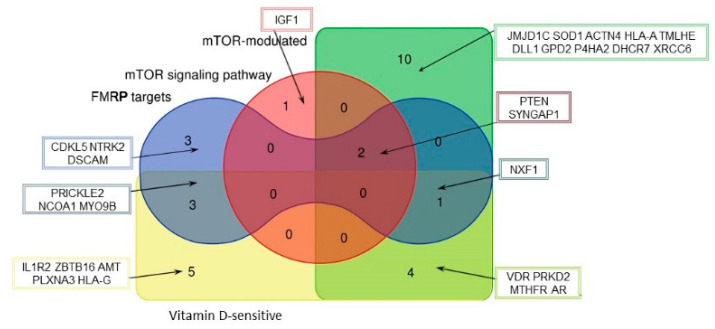
Venn diagram representing the complete intersection of ASD and AID genes of the four categories related to the mTOR signaling and vitamin D-sensitive genes.

We found that the intersection of 992 ASD and 871 AID was 54 genes, and 29 of them could be included in one of the four selected categories (Figure 3). Several genes that belonged to more than one category were of particular interest. We found that only three out of all ASD and AID genes fell into three categories simultaneously: PTEN (phosphatase and tensin homolog deleted on chromosome 10), SYNGAP1 (synaptic Ras GTPase activating protein 1), and NXF1 (nuclear RNA export factor 1). It is also worth mentioning that IGF1 (insulin-like growth factor 1) was the third one after PTEN and SYNGAP1 belonging to mTOR signaling and affected both ASD and AID.

### 2.2. AID Genes Network Analysis

To obtain more characteristics of the remaining 431 out of 871 AID genes that were not connected with mTOR signaling, FMRP, and vitamin D directly, we reconstructed a network presenting the interactions between these 431 genes and mTOR signaling and mTOR-modulated genes.

The initial set of 729 genes (27 AID mTOR signaling pathway genes, 271 AID mTOR-modulated genes, 431 AID non-categorized genes) was analyzed by STRING tools. For the 0.7 (high confidence) cutoff, we found 3124 interactions between 528 genes.

**Figure 4:**
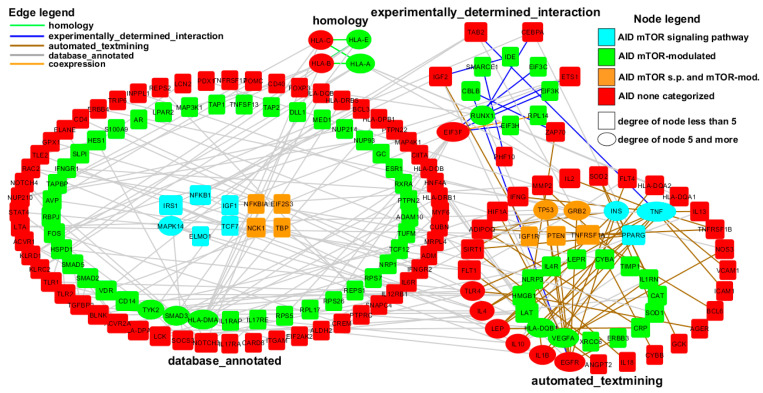
mTOR signaling and mTOR-modulated AID genes interacting with non-categorized AID genes. The right legend shows the color of the node description. The left legend shows the interaction type colors. The complete list of the most connected genes and their nodes degree is given in Supplementary Table S3.

For the 0.95 cutoff, the network contained 177 genes and 202 interactions (i.e., network edges) between them. Each edge colored according to the type of interaction made the greatest contribution to the combined score. There were five dominant types of interactions: annotated database (colored gray), automated textmining (colored brown), experimentally determined (colored blue), homology (colored green), and coexpression (just one interaction, colored orange) (Figure 4).

The genes were divided into groups according to the type of interaction. For the genes linked by several different types simultaneously, in our network, the priority of the gene location was given to a group less saturated with genes. The size of the gene node was set according to the number of their connections—the more interactions a gene was involved in, the larger the node size in the network. The most connected nodes of the network (with a node degree of 5 or more) were additionally converted to ellipses for better visual selection. Within each block, the genes were categorized according to whether they belonged to the AID mTOR signaling pathway (colored blue) or AID mTOR-modulated (colored green) category, both categories above simultaneously (colored orange), or the AID non-categorized category (colored red) (Figure 4).

Thus, we suggested that the overwhelming majority (up to 67% for the 0.7 cutoff) of AID genes are associated with the mTOR signaling pathway directly or indirectly.

Unsurprisingly for AID, among the most connected genes of the network (with a node degree of 5 or more), cytokine (TNF, IL 1B, IL 10, IL 4) and human leucocyte antigen (HLA-DMA, HLA-DQB1, HLA-A, HLA-B, HLA-C, HLA-E) genes were widely represented (Figure 4).

## 3. Discussion

Immune system abnormalities and autoimmunity have been frequently reported in children with ASD, reviewed in [20]. A combined family history of autoimmune disorders increased the risk of ASD by 28%, with the most significant increased risks associated with psoriasis (59%), rheumatoid arthritis (51%), type 1 diabetes (49%), and hypothyroidism (64%) [21]. The idea that autism might be an autoimmune disorder was discussed several times [20,22,23]. It was recently supported by the findings of the prominent, perivascular lymphocytic infiltrates and associated astrocyte blebs within the Virchow–Robin and subarachnoid cerebrospinal fluid (CSF) spaces in ~65% of ASD compared to control brains across a wide range of ages (5–68 years). The found neuropathology suggests that dysregulated cellular immunity damages astrocytes at foci along the CSF–brain barrier in ASD [24].

Previously, we conducted gene-set analyses and revealed that 606 out of 1053 genes (58%) included in the SFARI Gene database and 179 out of 281 high-confidence genes (64%) of the database could be attributed to one of the four groups: 1. FMRP target genes, 2. mTOR signaling network genes, 3. mTOR-modulated genes, and 4. vitamin D3-sensitive genes [19]. Here, we have demonstrated that with the exception of FMRP targets, that are obviously associated with the direct involvement of synaptic translation disturbance in the pathological mechanism of ASD, the remaining categories are represented in a very similar percentage among ASD- and AID-associated genes. Thus, mTOR signaling pathway genes make up 4% of ASD and 3% of AID genes, mTOR-modulated genes—31% of both ASD and AID genes, and vitamin D-sensitive genes—20% of ASD and 23% of AID genes.

None out of 992 ASD genes and 871 AID genes fell into all four examined categories, but 54 genes that associated with autism and autoimmune disorders simultaneously were of particular interest, with the largest number of intersections observed for mTOR-modulated genes (Figure 3). We found that only three out of all both ASD and AID genes fell into three categories simultaneously: PTEN (phosphatase and tensin homolog deleted on chromosome 10), SYNGAP1 (synaptic Ras GTPase activating protein 1), and NXF1 (nuclear RNA export factor 1). It could be suggested that such a versatile regulation makes these genes good candidates as drug targets for mechanism-based therapy.

PTEN is an important negative regulator of the AKT/mTOR signaling pathway; neurologically, heterozygous PTEN variants are associated with macrocephaly and syndromic autism (PTEN hamartoma tumor syndrome—PHTS) [25]. At the same time, PTEN mutations in humans and mice are associated with a skewed T- and B-cell gene repertoire, characterized by increased prevalence of high-frequency clones. Immunological characterization showed that PTEN mutants have increased B-cell proliferation and a proclivity towards increased T-cell reactivity upon Toll-like-receptor stimulation. Furthermore, germline disruption of PTEN, both in humans and mice, results in compromised central immune tolerance processes that may significantly affect individual stress responses and therefore predisposition to autoimmunity and cancer [25].

Mutations of the SYNGAP1 gene were first identified in 2009 in patients with intellectual disability (ID) and autism spectrum disorder (ASD), followed in 2013 by recognition of their important role in the developmental and epileptic encephalopathies (DEEs) [26]. It was unexpected to find the gene among AID predisposition genes. However, it was shown that low- and high-glycemic index (GI) diets differentially alter the levels of brain proteins involved in inflammation and synaptic function. The levels of SynGAP in the brains slightly differed between mice fed the two different diets [27].

The highly conserved protein nuclear RNA export factor 1 (Nxf1) is an important mediator of mRNA export from the nucleus to the cytoplasm [28]. Based on two “omics” approaches, NXF1 was identified as one of the IRF5 signaling pathway regulators, and in turn genome-wide association studies have implied the association of IRF5 with several autoimmune diseases, including SLE, Sjogren’s syndrome, inflammatory bowel disease, and MS [29]. Given a critical role for FMRP in the control of mRNA stability and for NXF1 in mRNA export from the nucleus, it becomes clear that the coordinated functioning of FMRP and NXF1 is necessary for normal local translation and synapse development [30].

The most abundant category of both ASD and AID candidate genes was the mTOR-modulated (Table 1). Recent studies showed that mTOR pathway activation plays critical roles in the pathogenesis of autoimmune diseases, including RA, immune thrombocytopenia, T1D, large-vessel vasculitis, and SLE. mTOR activity leads to Th1 and Th17 cell proliferation, Treg depletion, macrophage dysfunction, and increased antibody and immune complex production, ultimately resulting in tissue inflammation [31]. It is obvious that any mTOR-modulated gene expression would be disturbed under the mTOR pathway hyperactivation, that in turn would lead to elevated expressivity and/or penetrance of mTOR-modulated gene mutations for both ASD and AID.

Vitamin D-sensitive was the next most abundant category of AID and ASD genes (Table 1). A literature review demonstrated an inverse association between vitamin D and the development of several autoimmune diseases, such as SLE, thyrotoxicosis, T1D, MS, iridocyclitis, CD, UC, psoriasis vulgaris, seropositive RA, and polymyalgia rheumatic [32]. At the same time, higher serum concentrations of this steroid may reduce the risk of autism and ASD children are at an increased risk of vitamin D deficiency, possibly due to environmental factors. It has also been suggested that vitamin D3 deficiency may cause ASD symptoms [33]. Clinical trials, including case reports and case–control studies, have suggested that high-dose vitamin D3 regimens may ameliorate the core symptoms of ASD and some AID, including MS [33,34,35,36].

The network analysis revealed 3124 interactions between 528 out of 729 genes (27 AID mTOR signaling pathway genes, 271 AID mTOR-modulated genes, 431 AID non-categorized genes) for the 0.7 cutoff. It means that the vast majority (up to 67%) of AID genes are related to the mTOR signaling pathway directly or indirectly. The most connected gene of the network with a node degree of 12 was TNF (tumor necrosis factor). TNF is a cytokine that can bind to TNF receptor 1 (TNFR1) or TNF receptor 2 (TNFR2) and is involved in inflammation and the immune response [37]. TNF-alpha is elevated in psoriasis, RA, psoriatic arthritis, JRA, and ankylosing spondylitis [38]. It is worth mentioning that among the most connected genes of the network, cytokine (TNF, IL 1B, IL 10, IL 4) and human leucocyte antigen (HLA-DMA, HLA-DQB1, HLA-A, HLA-B, HLA-C, HLA-E) genes were plentiful (Figure 4).

Our present research and available published data allow us to hypothesize that both a certain part of ASD and AID comprise a connected set of disorders sharing a common aberrant pathway (mTOR signaling) rather than a vast set of different disorders. Furthermore, the immune subtype of the autism spectrum might be a specific type of autoimmune disorder with an early manifestation of a unique set of predominantly behavioral symptoms. Thus, following treatment with intravenous immunoglobulin (IVIG), significant improvement was observed in ASD children across several subscales of clinical tests and significant reductions were seen in the markers of neuroinflammation [39]. An environmental model of neurodevelopmental disorders in which mice were exposed to maternal immune activation (MIA) during embryogenesis has been compared with mouse models that were genetically deficient for Cntnap2, Fmr1, or Shank3. It was established that the social behavior deficits in offspring exposed to MIA could be temporarily rescued by the inflammatory response elicited by the administration of lipopolysaccharide (LPS). This behavioral rescue was accompanied by a reduction in neuronal activity in the primary somatosensory cortex dysgranular zone, the hyperactivity of which was previously implicated in the manifestation of behavioral phenotypes associated with offspring exposed to MIA. By contrast, there was no an LPS-induced rescue of social deficits in the monogenic models [40]. The efficacy and safety of prednisolone as an adjunctive treatment to risperidone was evaluated in children with regressive ASD, and it was shown that prednisolone, as an add-on to risperidone, could remarkably improve core features in children with regressive ASD [41].

Practically, the hypothesis of an immunological etiology of autism spectrum disorder could have a prospective translational effect of a clinical pathway for patients with new-onset ASD. In order to develop promising immune-based therapeutic approaches for autism and other psychiatric diseases, new diagnostic pathways have been proposed. In this proposed algorithm, individuals who present with new neuropsychiatric symptoms, such as ASD and psychosis, must be screened for blood, CSF, and imaging markers of inflammation [42]. If there are any signs of active inflammation, immunomodulatory treatment should be considered.

## 4. Materials and Methods

### 4.1. Extracting Genes from Diverse Data Sources

We analyzed the gene sets from the SFARI Gene database [1], KEGG database [43], and from seven published studies containing: 1. genes that are most reproducibly recognized as FMRP targets [44,45], 2. mTOR-sensitive genes from the NanoCAGE dataset [46], 3. genes included in the mTOR signaling network [47], 4. vitamin D-responsive genes and elements [48], and 5. genes connected with multiple sclerosis (MS), systemic lupus erythematosus (SLE), juvenile rheumatoid arthritis (JRA), Crohn’s disease (CD), ulcerative colitis (UC), and type 1 diabetes (T1D) [17,18]. Thus, six sets of genes were identified, and the set theory relations between them were examined in the work:

1. Genes implicated in autism susceptibility (from SFARI Gene database released 12-11-2020 (Supplementary Table S1))—992 genes;
2. FMRP target genes (1-s2.0-FMRP_tags_842-mmc2.xls [45]—842 genes and Jansen2017.xlsx [44]—1047 genes)—1614 genes;
3. Genes included in the mTOR signaling network (Table S1.xlsx [47]—248 genes and KEGG database—153 genes)—341 genes;
4. mTOR-sensitive genes (mTOR-sensitive 5UTR.xlsx [46])—6543 genes;
5. Vitamin D-responsive genes and elements (wang1.xls [48]—902 genes, wang5.xls [47]—3212 genes/loci)—3958 genes/loci;
6. Genes implicated in autoimmune disorders (2013.Tuller et al.S2.3-ANOVA.xlsx [18]—94 genes; 2013.Tuller et al.S2.3-PPI.xlsx [18]—109 genes; 2013.Tuller et al.S4.xlsx [18]—94 genes; 251_2020_1177_MOESM1_ESM.doc [17]—750 genes). In total—871 genes implicated in autoimmune disorder.

SFARI Gene 2.0 was used because it includes explicitly defined scoring criteria, continuous updates, and infrastructure to permit community-based involvement [1]. A stringent set of 842 FMRP target transcripts was identified with both antibodies (100%), using both CLIP protocols (100%) and different sequencing platforms (100%), and were biologically reproducible (99% were detectable in at least six of seven experiments) [45]. In addition, we included the gene set consisting of FMRP target genes (number of genes 1047), as defined by [44], that partially overlapped with a previous one [45]. A total of 248 genes encoding unique proteins were extracted from the most comprehensive map of the mTOR signaling network [47], and 153 genes were added to the group from the KEGG PATHWAY database, which is a collection of manually drawn pathway maps [43]. Transcription start site profiling using nano-cap analysis of gene expression (nanoCAGE) and ribosome-profiling allowed us to extract several types of mTOR-modulated mRNAs: 1. TOP (terminal oligopyrimidine motif) and TOP-like mRNAs via LARP1, 2. mRNAs with short 50 UTRs enriched for mitochondrial functions, which require EIF4E but are less EIF4A1-sensitive, and 3. long 50 UTR mRNAs encoding proliferation- and survival-promoting proteins, which are both EIF4E- and EIF4A1-sensitive [46]. In our analysis, two sets of vitamin D-modulated genes were used: 1. 1.25(OH)2D3 target genes identified by screening Affymetrix Hu133A Oligonucleotide Microarrays, 2. genes identified by genome screening bearing consensus VDREs or DR3 elements with single-nucleotide substitutions and including elements as being conserved between human and mouse, even if they differed in VDRE sequence [48]. To create a set of genes implicated in autoimmune disorders we combined two gene collections: genes over/underexpressed in six major autoimmune diseases that were consolidated and scored by [18], and meta-analyses-based T1D-associated genes [17].

### 4.2. Assignment of Genes to Categories and Pathway Analysis

To assign the genes implicated in autism spectrum disorders (ASD) and the genes associated with autoimmune disorders (AID) into the categories related to the mTOR signaling pathway and vitamin D-regulated genes, we performed a gene-set analysis and constructed the Venn diagrams that are widely used in bioinformatics as a tool for gene-set analysis [49,50].

Therefore, the first step was to obtain lists of genes belonging to one of the categories 2–5, which at the same time were among the autism-related genes from the SFARI Gene database. The second step was to split the joint set of over/underexpressed in six major autoimmune diseases genes and T1D-associated genes into the same categories. We then analyzed the resulting lists, highlighting the ASD genes that appeared in more than one category as well as the AID genes and the intersections between these two sets.

Customized Python scripts were used to perform the comparison of lists of gene sets and calculate intersections and complements (see Supplementary Table S2). As a key for comparison, a unique identifier “gene symbol” assigned to each of the *Homo sapiens* genes was used. To take into account possible synonymous gene names, we used the KEGG synonym table (http://rest.kegg.jp/list/hsa accessed on 18 December 2020) to convert genes symbols into NCBI gene IDs that were used for the consequent comparison. We used the service http://bioinformatics.psb.ugent.be/webtools/Venn/ for constructing Venn diagrams.

### 4.3. Network Construction

The next step was to reconstruct the gene network using STRING (Search Tool for the Retrieval of Interacting Genes/Proteins) [51] and Cytoscape [52] tools. STRING allows building a network based on a unique list of genes, adding various types of interactions as edges (for example, found by the textmining procedure, in the annotated databases, or experimentally determined, etc.). Each individual interaction had a score that ranged from 0 to 1; the higher the value, the more reliable the relationship. Based on these scores, the final combined score was formed that determined whether the interaction would be included in the resulted network. To search for interactions, one can specify various confidence options (medium confidence (0.4), high confidence (0.7), highest confidence (0.9)). Cytoscape allows us to visualize the network based on the found interaction data. Thus, the reconstruction procedure consisted of the following stages:

1. The initial set of 729 genes (27 AID mTOR signaling pathway genes, 271 AID mTOR-modulated genes, 431 AID non-categorized genes) was submitted to the STRING database, the cutoff for interactions was 0.7 (high confidence). At the first stage, we found 3124 interactions between 528 genes;
2. Based on the found interactions, the network was reconstructed in the Cytoscape software package. Since we were mainly interested in the links between AID mTOR-dependent genes and AID non-categorized genes, the links within these groups were excluded, which allowed us to reduce the number of genes to 421, and the number of interactions to 1124;
3. We increased the confidence cutoff from 0.7 to 0.9 and then to 0.95. The network for each of the above cutoffs is available in Supplementary Figure S1 (network.cys).

## Figures and Tables

**Table 1:** A generalized table representing the relationships of four categories of genes: FMRP (fragile X mental retardation protein) target, mTOR (mechanistic target of rapamycin) signaling network, mTOR-modulated, and vitamin D-sensitive genes for SFARI (Simon’s Foundation Autism Research Initiative) Gene database and AID gene set.

Category of Genes	Number of Genes		
	SFARI (Whole)	Autoimmune Disorders	SFARI (High Confidence + Strong Candidate)
FMRP target	270	74	146
mTOR signaling pathway	41	27	18
mTOR-modulated	304	271	134
Vitamin D-sensitive	202	199	78
None	408	431	143
Total	992	871	401

## Supplementary Materials

The following are available online at https://www.mdpi.com/article/10.3390/ijms22105248/s1, Figure S1: mTOR-AID network, Table S1: SFARI-Gene_genes_12-11-2020 release, Table S2: SFARI Gene database and AID genes divided, Table S3: Degree of nodes.

## Abbreviations

ASD	autism spectrum disorder
AID	autoimmune disorders
SFARI	Simon’s Foundation Autism Research Initiative
mTOR	mechanistic target of rapamycin
FMRP	fragile X mental retardation protein
